# Predictive value of the neutrophil to lymphocyte ratio for disease deterioration and serious adverse outcomes in patients with COVID-19: a prospective cohort study

**DOI:** 10.1186/s12879-021-05796-3

**Published:** 2021-01-18

**Authors:** Zhi-Yong Zeng, Shao-Dan Feng, Gong-Ping Chen, Jiang-Nan Wu

**Affiliations:** 1 Department of Hematology, the First Affiliated Hospital of Fujian Medical University, Fuzhou, China; 2 Department of Emergency, the First Affiliated Hospital of Fujian Medical University, Fuzhou, China; 3 Department of Respiratory and Critical Care Medicine, the First Affiliated Hospital of Fujian Medical University, 20 Chazhong Rd., Fuzhou, 300005 China; 4 Department of Clinical Epidemiology, Obstetrics and Gynecology Hospital of Fudan University, 566 Fangxie Rd., Shanghai, 200011 China

**Keywords:** Neutrophil to lymphocyte ratio, Deterioration, Shock, Death, COVID-19

## Abstract

**Background:**

Early identification of patients who are at high risk of poor clinical outcomes is of great importance in saving the lives of patients with novel coronavirus disease 2019 (COVID-19) in the context of limited medical resources.

**Objective:**

To evaluate the value of the neutrophil to lymphocyte ratio (NLR), calculated at hospital admission and in isolation, for the prediction of the subsequent presence of disease progression and serious clinical outcomes (e.g., shock, death).

**Methods:**

We designed a prospective cohort study of 352 hospitalized patients with COVID-19 between January 9 and February 26, 2020, in Yichang City, Hubei Province. Patients with an NLR equal to or higher than the cutoff value derived from the receiver operating characteristic curve method were classified as the exposed group. The primary outcome was disease deterioration, defined as an increase of the clinical disease severity classification during hospitalization (e.g., moderate to severe/critical; severe to critical). The secondary outcomes were shock and death during the treatment.

**Results:**

During the follow-up period, 51 (14.5%) patients’ conditions deteriorated, 15 patients (4.3%) had complicated septic shock, and 15 patients (4.3%) died. The NLR was higher in patients with deterioration than in those without deterioration (median: 5.33 vs. 2.14, *P* < 0.001), and higher in patients with serious clinical outcomes than in those without serious clinical outcomes (shock vs. no shock: 6.19 vs. 2.25, *P* < 0.001; death vs. survival: 7.19 vs. 2.25, *P* < 0.001). The NLR measured at hospital admission had high value in predicting subsequent disease deterioration, shock and death (all the areas under the curve > 0.80). The sensitivity of an NLR ≥ 2.6937 for predicting subsequent disease deterioration, shock and death was 82.0% (95% confidence interval, 69.0 to 91.0), 93.3% (68.0 to 100), and 92.9% (66.0 to 100), and the corresponding negative predictive values were 95.7% (93.0 to 99.2), 99.5% (98.6 to 100) and 99.5% (98.6 to 100), respectively.

**Conclusions:**

The NLR measured at admission and in isolation can be used to effectively predict the subsequent presence of disease deterioration and serious clinical outcomes in patients with COVID-19.

**Supplementary Information:**

The online version contains supplementary material available at 10.1186/s12879-021-05796-3.

## Background

The worldwide novel coronavirus disease 2019 (COVID-19) pandemic has caused great loss of lives. According to the WHO COVID-19 dashboard, as of November 24, 2020, approximately 1.4 million patients died out of a total of > 58 million confirmed cases, with an average mortality of 2.40%. Mortality was positively correlated with the severity of the disease. A summary of a report of 72,314 cases in China indicated that the case-fatality rate was as high as 49% in critical cases, while the average mortality in all confirmed cases was 2.3% [1]. Therefore, early identification of patients with COVID-19 who are at high risk of poor clinical outcomes is of great importance [2].

The neutrophil to lymphocyte ratio (NLR), whether measured in isolation or in combination with other risk factors, has been identified as a useful marker in distinguishing severe cases from mild/moderate cases and is associated with COVID-19 mortality [3–9]. However, little is known about the value of the NLR in terms of predicting the clinical condition of the disease (e.g., mild/moderate progression to severe/critical, severe deterioration to critical) and serious clinical outcomes (e.g., shock, death). We conducted a prospective cohort study to evaluate the value of using the NLR measured at hospital admission to predict the subsequent presence of poor clinical outcomes.

## Methods

### Study design and data collection

We conducted a prospective cohort study of COVID-19 patients who received medical treatment at the Third People’s Hospital of Yichang, Hubei Province, from January 9 to February 26, 2020. All patients who were confirmed by laboratory tests (positive for RT-PCR of nasal and pharyngeal swab specimens and/or specific IgM/G antibody assay of serum) before admission were recruited for the study. Basic information (e.g., age, sex, smoking, drinking, and history of comorbidities) was collected through face-to-face interviews with the patients or their families using a standardized questionnaire [2]. Routine blood examinations, including complete blood cell counts, coagulation profiles and serum biochemical tests, were conducted at hospital admission. Patients with missing neutrophil and/or lymphocyte records at hospital admission were excluded from the study. The patients were then treated according to the diagnosis and treatment plan for COVID-19 (7th version) issued by the National Health Commission of the People’s Republic of China [10] and were followed until discharge (for those who recovered) or death. The last patient was discharged on March 25, 2020. The changes in disease condition, treatment details and clinical outcomes of the disease were recorded during the follow-up period. The study was approved by the ethics committee of the First Affiliated Hospital of Fujian Medical University. The hospital was in charge of supporting the treatment of patients with COVID-19 in Yichang City.

### Exposure and outcome

Patients with an NLR, calculated by the neutrophil and lymphocyte data measured at hospital admission, equal to or higher than the cutoff value (2.6937) were categorized as the exposed group; otherwise, they were classified as the control group. The COVID-19 condition at hospital admission was evaluated and classified into mild/moderate, severe, and critical according to the diagnosis and treatment guidelines [10]. Patients were reclassified if their condition worsened and reached a higher classification criterion.

The primary outcome was disease deterioration, defined as progression of disease severity during hospitalization, including changes from from mild/moderate to severe and/or critical and from severe to critical. The secondary outcomes were shock and death during the treatment.

### Data analysis

The NLR was expressed as the median (interquartile range) since the data were nonnormally distributed (*P* < 0.001 for Kolmogorov-Smirnov test). Categorical data are expressed as n (%) between patients with and without disease deterioration. Differences between the two groups were compared by chi-square or Fisher’s exact test (if applicable) for categorical variables. Box diagrams of the distribution of NLR between patients with and without the outcomes (i.e., disease deterioration, shock, death) are shown and were compared by Mann-Whitney *U*-test.

The predictive performance of the NLR for disease deterioration, shock and death was assessed by estimating the area under the curve and the corresponding 95% confidence intervals (95% CI) of the receiver operating characteristic curve method. The cutoff value and corresponding sensitivity, specificity, positive predictive value and negative predictive value were estimated.

The association of a higher NLR (defined as an NLR of ≥2.6937) with the risk of disease deterioration, shock and death was further estimated by a Cox proportional hazards model, in which potential confounders included maternal age (< 60 or ≥ 60 years), sex (male or female), smoking (yes or no), drinking (yes or no), clinical COVID-19 condition at admission (mild/moderate, severe, or critical) and history of chronic diseases (yes or no, defined as history of any one of hypertension, diabetes mellitus, coronary heart disease, cerebral vascular disease, chronic obstructive pulmonary disease or cancer). The Kaplan-Meier curve method was used to compare the cumulative risk of disease deterioration, shock and death between patients with an NLR above and below the cutoff value. Cases with missing data were excluded from the analysis mentioned above. All statistical tests were conducted using IBM SPSS Statistics version 22.0. A two-sided *P* value < 0.01 was considered statistically significant.

## Results

### Baseline characteristics

A total of 352 patients with COVID-19 were included in the cohort and followed until the outcome was observed (average follow-up time of 24.3 days, ranging from 5 to 51 days). Among these subjects, 341 patients (96.9%) had a measured NLR at hospital admission and were included in the analysis. The mean (SD) age of patients in the cohort was 52.1 (18.1) years; 190 patients (54.0%) were men, and 117 patients (33.2%) had a history of at least one comorbidity. At hospital admission, 301 patients (85.5%) were classified as mild/moderate, 48 (13.6%) were classified as severe and 3 (0.9%) were classified as critical. During the follow-up, 51 (14.5%) patients’ conditions deteriorated, 15 patients (4.3%) had complicated septic shock, and 15 patients (4.3%) died (Table S1). Baseline characteristics, except smoking and drinking, were found to be unbalanced between patients with and without deterioration (Table 1).

**Table 1:** Basic characteristics of the 352 patients with COVID-19

Characteristics	Disease deterioration		*P* value
	No (*N* = 301)	Yes (*N* = 51)	
NLR (median, inter-quartile range) ^a^	2.14 (1.53–2.98)	5.33 (2.94–9.72)	< 0.001
Age (≥60 years), n (%)	105 (34.9)	28 (54.9)	0.006
Male, n (%)	156 (51.8)	34 (66.7)	0.049
Smoking, n (%) ^a^	46 (15.3)	11 (21.6)	0.233
Drinking, n (%)	21 (7.0)	2 (3.9)	0.551 ^c^
History of chronic disease ^b^	83 (27.6)	34 (66.7)	< 0.001
Clinical type at admission			< 0.001 ^c^
Mild or moderate	282	19	
Severe	16	32	
Critical	3	0	
Shock ^a^	0 (0)	15 (30.6)	< 0.001 ^c^
Death	0 (0)	15 (29.4)	< 0.001 ^c^

^a^ Sample sizes of the analysis for neutrophil to lymphocyte ratio, smoking and shock were 341, 351 and 349, respectively

^b^ Defined as history of any one of the following six diseases: hypertension, diabetes mellitus, coronary heart disease, cerebral vascular disease, chronic obstructive pulmonary disease and cancer

^c^ *P* value of Fisher’s exact test

### Predictive performance

**Fig. 1:**
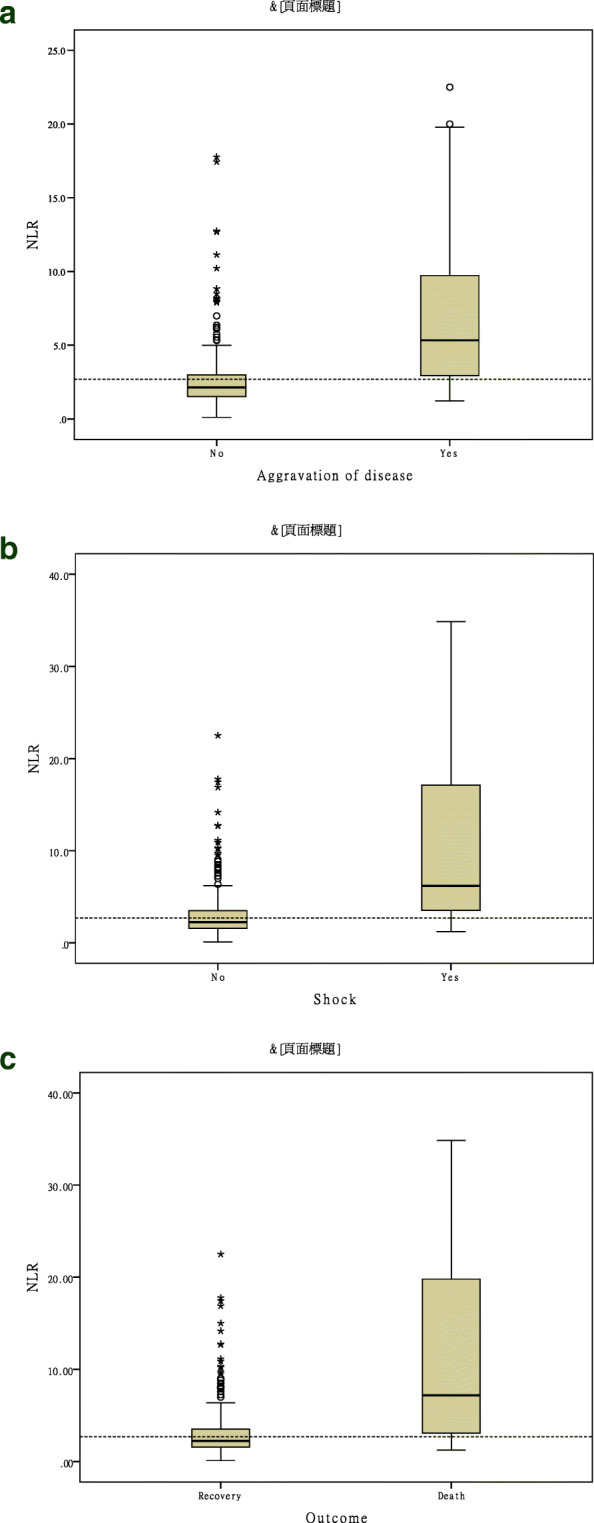
Neutrophil to lymphocyte ratio for patients with and without disease deterioration, shock and death. Disease deterioration (panel **a**), shock (panel **b**) and death (panel **c**). The bottom and top edges of each box represent the first (P25) and third quartiles (P75), respectively. The band within the box represents the median (P50), and the whiskers represent values that are 1.5 times the interquartile range. The horizontal line represents the cutoff value of 2.6937 for the NLR. Two extreme outliers (> 3 times the interquartile range), including a patient with an NLR of 85.52 in the groups without adverse outcomes and a patients with an NLR of 54.36 in the groups with adverse outcomes, were not shown. NLR represents the neutrophil to lymphocyte ratio

The median NLR measured at hospital admission was 2.30, with a range from 0.11 to 85.5. The NLR was higher in patients with deterioration than in those without deterioration (median: 5.33 vs. 2.14, *P* < 0.001), and higher in patients with serious clinical outcomes than in patients without serious clinical outcomes (shock vs. no shock: 6.19 vs. 2.25, *P* < 0.001; death vs. survival: 7.19 vs. 2.25, *P* < 0.001) (Table 1, Fig. 1).

**Fig. 2:**
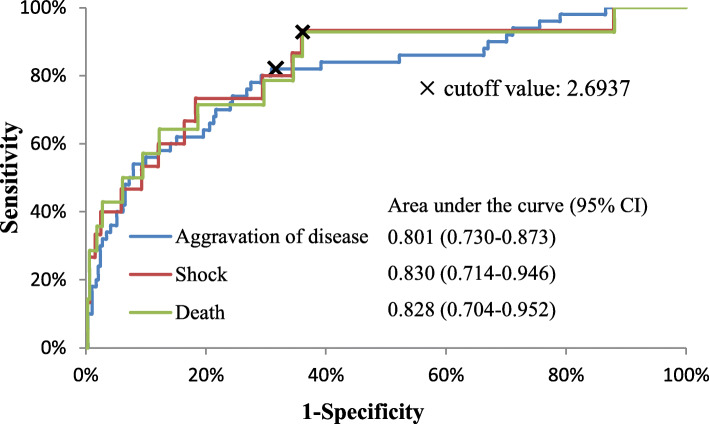
Receiver operating characteristic curves of neutrophil to lymphocyte ratios in predicting serious clinical outcomes in patients with COVID-19. CI represents the confidence interval

The NLR measured at hospital admission had high value in predicting disease deterioration, shock and death, with areas under the curve of 0.801, 0.830, and 0.828, respectively, and a cutoff value of 2.6937 (Fig. 2). The sensitivity of the ≥2.6937 NLR cutoff for predicting the subsequent presence of disease deterioration, shock and death was 82.0% (95% CI, 69.0 to 91.0), 93.3% (68.0 to 100), and 92.9% (66.0 to 100), respectively, and the corresponding negative predictive values were 95.7% (93.0 to 99.2), 99.5% (98.6 to 100) and 99.5% (98.6 to 100), respectively (Table 2).

**Table 2:** Validation of a cutoff value of 2.6937 for the neutrophil to lymphocyte ratio in predicting serious clinical outcomes for patients with COVID-19

Serious clinical outcomes	No. of patients	% (95% confidence interval)			
		Sensitivity	Specificity	Positive predictive value	Negative predictive value
Deterioration	341	82.0 (69.0–91.0)	69.1 (63.8–74.8)	31.3 (23.4–39.7)	95.7 (93.0–99.2)
Shock	338	93.3 (68.0–100)	64.1 (58.9–69.7)	10.8 (5.4–16.3)	99.5 (98.6–100)
Death	341	92.9 (66.0–100)	63.9 (58.7–69.5)	9.9 (4.8–15.2)	99.5 (98.6–100)

Compared with patients who had an NLR of < 2.6937, the adjusted hazard ratios for disease deterioration, shock and death were 4.1, 13.1 and 11.3, respectively, among those with an NLR of ≥2.6937 (Table S2). The cumulative risk of the clinical outcome was significantly higher in patients with an NLR of ≥2.6937 than in those with an NLR of < 2.6937 (all *P* < 0.001, Figure S1).

## Discussion

In this study, we validated a cutoff value of 2.6937 for the NLR, calculated at hospital admission, as a useful predictor of the subsequent presence of disease deterioration and the occurrence of serious clinical outcomes, such as shock and death. COVID-19 patients with an NLR of < 2.6937 had a possibility of ruling out disease deterioration (negative predictive value of 95.7%) and serious clinical outcomes (negative predictive value of 99.5%).

Early detection of patients whose condition is likely to worsen may aid in delivering proper care and optimizing the use of limited resources [2]. The NLR in the peripheral blood is related to the systemic inflammatory state and disease activity and shows prognostic value in cardiovascular diseases, autoimmune diseases, malignant tumours and infectious diseases [11–14]. For patients with COVID-19, in addition to severe lung lesions, prominent derangement of the lymph haematopoietic system has been noted [15]. An elevated NLR may reflect the severity of COVID-19 and the immune status of the patients.

Recently, some retrospective studies have identified the role of the NLR in discriminating severe patients and predicting the mortality of COVID-19 patients [3–8]. The NLR was also included as a variable in a clinical risk score to predict the occurrence of critical illness in hospitalized patients with COVID-19 [2]. The present study prospectively validates the findings of these previous studies through a large sample of 352 patients and extends the role of NLR in predicting disease deterioration and serious clinical outcomes, such as shock. Furthermore, the present study identified an NLR cutoff value of 2.6937, above which most patients’ (≥ 82.0%) condition worsened and serious clinical outcomes occurred; patients with an NLR about this cutoff (NLR ≥ 2.6937) were included in the high-risk group, and the negative predictive value (the possibility of ruling out the outcomes) of a ratio below the cutoff value was ≥95.7%. This finding may improve the accuracy of clinical decisions made at hospital admission among clinicians treating patients with COVID-19, especially in regions with high case volumes and/or limited medical resources.

The present study has some limitations. First, the sample size was large but might not have been large enough to evaluate the predictive performance of the NLR for shock and death since only 15 cases of shock and 14 deaths were included in the analysis in this cohort, resulting in wide of 95% confidence intervals for the sensitivity of shock and death. Second, we were not able to exclude the impact of some other treatments before hospital admission on the outcome, which may have resulted in a potential overestimation of the NLR performance. For example, the NLR may be affected by steroid therapy, especially in those with an NLR ranging from 5 to 8 [16]. However, the potential bias (if it exists) is slight and might not be enough to change the results since the possibility of steroid therapy before admission is rare, NLR was tested immediately at hospital admission, and only 20 patients (5.9%) were located in the sensitive range of 5.0–8.0 for the NLR. Finally, mortality of the disease may affect the performance of NLR in predicting the outcomes. Studies on the applicability of the results in populations with various COVID-19 mortalities are warranted.

## Conclusions

In conclusion, in this prospective cohort study of 352 patients with COVID-19, the NLR calculated at hospital admission had high value in predicting the subsequent presence of disease deterioration and serious clinical outcomes and may be worth promoting in areas battling the COVID-19 pandemic.

## Supplementary Information

**Additional file 1: Figure S1.** Cumulative risk of disease deterioration, shock and death between patients with ≥2.6937 and < 2.6937 for the neutrophil to lymphocyte ratio measured at admission. Disease deterioration (panel A), shock (panel B) and death (panel C).

**Additional file 2: Table S1.** Disease progression in 352 patients with COVID-19 ^a^.

**Additional file 3: Table S2.** Hazard ratios for serious clinical outcomes among COVID-19 patients with a neutrophil to lymphocyte ratio ≥ 2.6937 compared with those of < 2.6937.

## Data Availability

Data and materials were available from the corresponding authors (wjnhmm@126.com or cgp3542@163.com) on reasonable request.

## Abbreviations

CI: Confidence interval
COVID-19: Corona virus disease 2019
NLR: Neutrophil to lymphocyte ratio
