# Supplementary figures and images for: Predictive value of the neutrophil to lymphocyte ratio for disease deterioration and serious adverse outcomes in patients with COVID-19: a prospective cohort study
Source: BMC Infect Dis. 2021 Jan 18;21:80. doi: 10.1186/s12879-021-05796-3 (PMC7812552; doi:10.1186/s12879-021-05796-3)

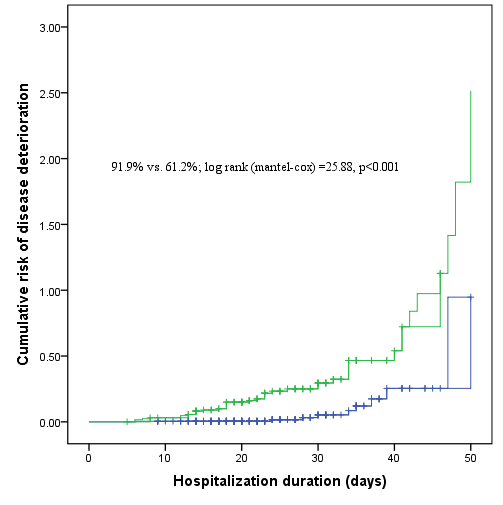


A


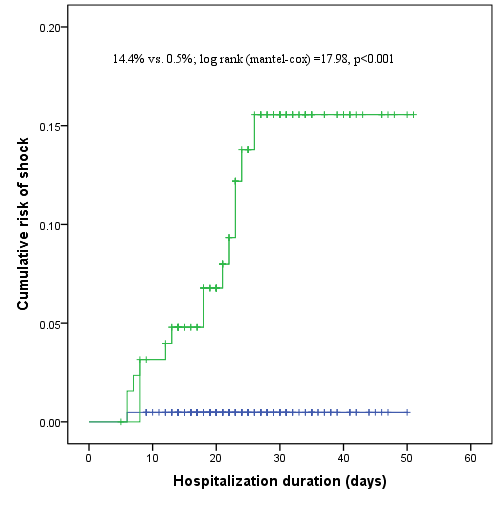


B


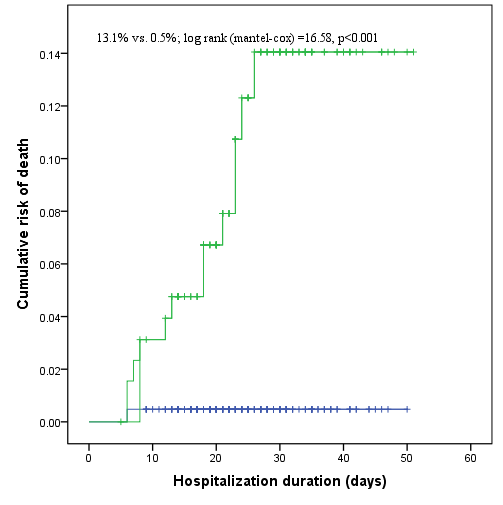


C

Figure S1.

Supplement: Supplementary file 1 — Additional file 1: Figure S1. Cumulative risk of disease deterioration, shock and death between patients with ≥2.6937 and < 2.6937 for the neutrophil to lymphocyte ratio measured at admission. Disease deterioration (panel A), shock (panel B) and death (panel C). [file 12879_2021_5796_MOESM1_ESM.docx]
