# Supplementary material for: Predictive value of the neutrophil to lymphocyte ratio for disease deterioration and serious adverse outcomes in patients with COVID-19: a prospective cohort study
Source: BMC Infect Dis. 2021 Jan 18;21:80. doi: 10.1186/s12879-021-05796-3 (PMC7812552; doi:10.1186/s12879-021-05796-3)
Supplement: Supplementary file 2 — Additional file 2: Table S1. Disease progression in 352 patients with COVID-19 a. [file 12879_2021_5796_MOESM2_ESM.docx]

Table S1. Disease progression in 352 patients with COVID-19 ^a^

| Clinical classifications at admission | | Final classifications during the treatment | | | |
| --- | --- | --- | --- | --- | --- |
|  |  |  | | | |
|  |  | Mild/moderate | Severe | Critical | Total |
|  | Mild/moderate | 282 | 18 (1) ^b^ | 1 | 301 |
|  | Severe | 0 | 17 | 31 (14, 15) ^b^ | 48 |
|  | Critical | 0 | 0 | 3 | 3 |
|  | Total | 282 | 35 | 35 | 352 |

^a^ P < 0.001 for the McNemar test.

^b^ One shock occurred in the 18 severe patients who progressed from mild/moderate, while 14 shocks and 15 deaths occurred among the 31 critical patients who developed from severe conditions.
