# Supplementary material for: Predictive value of the neutrophil to lymphocyte ratio for disease deterioration and serious adverse outcomes in patients with COVID-19: a prospective cohort study
Source: BMC Infect Dis. 2021 Jan 18;21:80. doi: 10.1186/s12879-021-05796-3 (PMC7812552; doi:10.1186/s12879-021-05796-3)
Supplement: Supplementary file 3 — Additional file 3: Table S2. Hazard ratios for serious clinical outcomes among COVID-19 patients with a neutrophil to lymphocyte ratio ≥ 2.6937 compared with those of < 2.6937. [file 12879_2021_5796_MOESM3_ESM.docx]

Table S2 Hazard ratios for serious clinical outcomes among COVID-19 patients with a neutrophil to lymphocyte ratio ≥ 2.6937 compared with those of < 2.6937

| Disease progression | Hazard Ratio (95% confidence interval) | |
| --- | --- | --- |
|  | Unadjusted | Adjusted ^a^ |
| Deterioration | 5.4 (2.6-11.1) | 4.1 (1.9-8.8) |
| Shock | 21.2 (2.8-161.3) | 13.1 (1.7-102.3) |
| Death | 19.8 (2.6-151.4) | 11.3 (1.4-89.1) |

^a^ Age (< or ≥ 60 years), sex (male or female), smoking (yes or no), drinking (yes or no), clinical classifications at admission (mild/moderate, severe, or critical) and history of chronic diseases (yes or no) were included in the adjusted model.
